# Configuration of Novel Experimental Fractographic Reverse Engineering Approach Based on Relationship between Spectroscopy of Ruptured Surface and Fracture Behaviour of Rubber Sample

**DOI:** 10.3390/ma13194445

**Published:** 2020-10-07

**Authors:** Sanjoy Datta, Radek Stoček, Evghenii Harea, Ondřej Kratina, Martin Stěnička

**Affiliations:** Centre of Polymer Systems, Tomas Bata University in Zlín, tř. Tomáše Bati 5678, 760 01 Zlín, Czech Republic; stocek@utb.cz (R.S.); harea@utb.cz (E.H.); okratina@utb.cz (O.K.); stenicka@utb.cz (M.S.)

**Keywords:** rubber, fractography, uniaxial tensile, deformation speed, fracture, tearing energy, oil, spectroscopy

## Abstract

A novel fractographic approach based on a combination of (i) mechanical behavior of cured rubber in uniaxial tensile loading and (ii) spectroscopy of fracture on a ruptured surface was experimentally validated. This approach related the migration of paraffin oil from a matrix to the ruptured rubber surface, to the tearing energy related to the deformation speed responsible for total rubber sample rupture, and the approach itself was configured experimentally. It was evaluated on cured natural rubber (NR) for two different paraffin oil concentrations. Single edge notched tensile (SENT) samples were subjected to uniaxial tensile loadings at two different deformation speeds. First, the tearing energy as a function of deformation speed was determined for each defined oil concentration. Secondly, at specific locations on the ruptured surfaces, infrared (IR) spectroscopy was performed to quantify a characteristic absorbance peak height of migrated paraffin oil during the rupture process. The results of the IR analyses were related to the deformation speed to understand the relation between the amount of migrated paraffin oil during the fracture process and the deformation speed which brought about such a fracture. This novel approach enhanced the reverse engineering process of rubber fracture related to the cause of tearing energies during critical failure.

## 1. Introduction

Failure of structural components is mostly due to the initiation of micro-cracks and their propagating in macroscopic scale. Since in real material defects naturally are present as precursors of macroscopic cracks, the fracture mechanic is used to understand the material behavior due to propagation of a macroscopic crack, or even to do fractographic analyses of a ruptured surface after the material failure. The increase of fracture resistance by modifying the structure of materials is an important objective of materials science not only for structural materials such as steel, but also for highly deformable rubbery materials. Therefore, improving the crack growth resistance of rubber materials has been a very important goal in the rubber industry for a long time [1,2,3].

Rubbery materials are not often used for contemplated products unless they are properly compounded and cured. The compounding of rubber is done to optimize properties to meet a given service application or set of performance parameters. Some of the more important compounding ingredients used are cross-linking and curing agents, reinforcing fillers, anti-degradants, processing aids, plasticizers or extenders and tackifiers [4], existing as either rigid solid or liquid matter in the rubber matrix.

Generally, the ingredients based on liquid matter are able to migrate in the volume of the rubber matrix with respect to the curing network, the migration being powered by the stress concentration caused due to deformation of the rubber matrix under loading. One of the most used liquid ingredients is oil, acting as an extender in rubber compounding. It aids in the incorporation of much higher amounts of reinforcing fillers in rubbers, especially in rubbers with higher molecular weights [4].

**Figure 1:**
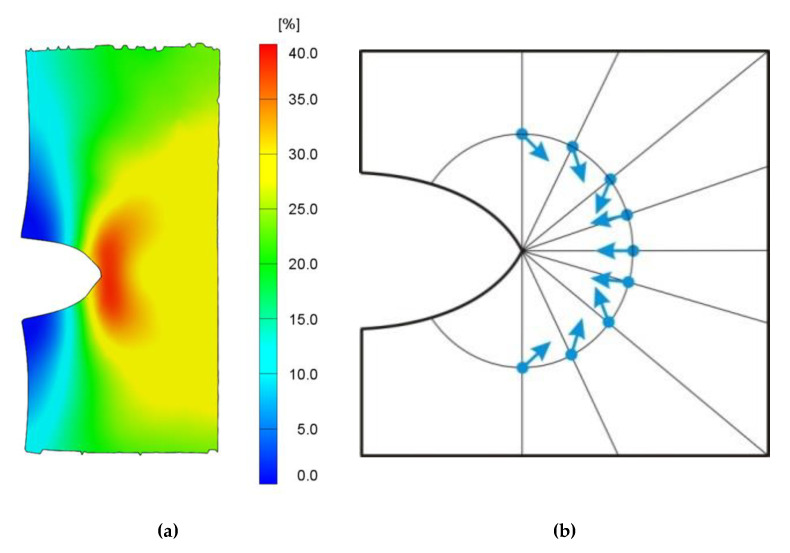
Tensile deformation of an oil incorporated rubber sample in vertical direction visualized via (**a**) major strain (horizontal direction); (**b**) a scheme showing volume force orientation initially shown in the work of Kaliske et al. [5].

Evaluated strain fields of an oil incorporated single edge notched tensile (SENT) rubber sample from digital image correlation (DIC) analyses is shown in Figure 1a, where a pronounced crack blunting can be observed during the classical tensile test (in uniaxial vertical direction) and subsequently calculated via software GOM Snap 2D and GOM Correlate (GOM GmbH, Braunschweig, Germany). As expected, the highest deformation was just behind the notch tip, which was due to the vertical deformation, distributed above and below the tip, creating a bean-shaped area. At farther distance behind the notch tip, the deformation gradually decreased. The results are in very good agreement with the numerical calculation published by Kaliske et al., Figure 1b [5]. Based on publication [5], the results of numerical simulation of the material forces are shown as nodal vectors. The force vectors are composed by the driving forces on the plastic zone and the driving forces on the crack tip. As is obvious, they are concentrated around the crack tip and their orientation causes the crack opening, which in turn causes fracture. In addition, the forces are acting on the solid as well as on liquid matter in the matrix in a similar manner and in the same direction. This means that the gradients are oriented in the direction of the crack tip.

Thus, it is assumed that the liquid matter would be relatively extracted onto the fracture surface from the matrix after the incidence of fracture. The relative change in the amount of the liquid matter on such a surface after a fracture has been enhanced is dependent on the viscoelastic property of the solid matter, the viscous property of the liquid matter, the loading condition on the whole material and the respective stress concentration in the vicinity of the crack tip.

Considering the process of incremental crack propagation in a solid causing fracture, the global energy balance under quasi-static state according to Equation (1) reads:

(1) $$\frac{dW_{ext}}{dA}-\frac{dW}{dA}=\frac{dW_{sep}}{dA}+\frac{dW_{diss}}{dA}$$

where *W_ext_* is the external work, *W* is the recoverable elastic strain energy, *W_diss_* is the dissipated energy and *W_sep_* is the local work of separation. In the case of a plane specimen with a constant thickness, *B,* and a straight crack front, the increment of the cracked area is *dA = Bda* with *da* being the crack length increment [6,7].

Considering the external work is zero, i.e., the clamp distance, *l*, is constant during the process of crack propagation, the tearing energy, *T*, can be derived as is shown in Equation (2).

(2) $$T=-{\frac{dW}{dA}|}_{l=const}=-{\frac{dW}{Bda}|}_{l=const}$$

However, energy dissipation mechanisms in rubber are not restricted to the immediate vicinity of the crack front. Moreover, these dissipation mechanisms can be rate-dependent and rate-independent, and are not only due to the crack propagation, but also to specific loading conditions [8].

Many works related to the calculation of tearing energy using single edge notched tensile (SENT) specimens are reported in the literature [9,10,11,12,13]. Researchers have tried to precisely calculate the energy release rate by using the J-integral [9,10,11,14,15,16].

**Figure 2:**
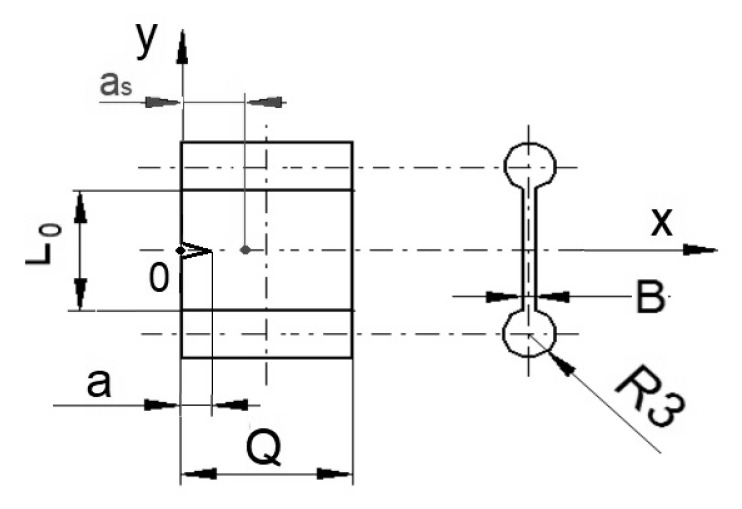
Geometry of single edge notched tensile (SENT) specimen.

Rice et al. [17] showed that instead of using the energy balance, the J-integral can be more easily determined directly from the force, F – deformation, l curve by using the approximate formula shown in Equation (3).

(3) $$J=\frac{\eta \cdot W_{def}}{B\cdot (Q-a)}$$

with *η* being a dimensionless geometry function of the crack length, a to specimen width ratio *a/Q* of the SENT sample (Figure 2). However, it should be noted that for the considered very limited amount of stable crack propagation in filled elastomer, the geometry function can be assumed to be constant. The deformation energy, *W_def_*, was determined by integrating the F–l diagram up to certain deformation values.

Here, the dimensions of length *l_0_* = 100 mm, width *Q* = 25 mm and thickness *B* = 6 mm and *a/Q* ratio of at least 0.2 were preferred [9,18].

The literature survey reveals that tests performed using SENT specimens were very specific to the calculation of the tearing energy based on the phenomenon of the additional area generated during the process of tear, and they were calculated on predetermined specimens of given dimensions. However, these works did not postulate the way to reconstruct the history of rupture after the incidence of the rupture took place, e.g., the relative speed of deformation of a specimen, which brought about the rupture.

Thus, this idea evolved that once the fracture process proceeds and the new crack surface is generated, broad fractographic investigations may be performed to reverse engineer the complete fracture process to understand the reason causing the mechanism. This may be applicable to both a totally ruptured sample, as well as a partly cracked sample, causing a local fracture instead of total rupture. The fractographic investigations of rubber material were mainly based on a varied microscopic investigation of the fractured surface topology [19,20].

However, a literature study reveals that until now, and apart from the microscopic investigations, very limited investigations related to fractography have been performed to reverse engineer a fracture process. Ducrot et al. [21] studied the fracture surfaces by the use of mechano-fluorescence spectroscopy. There are no other fractographical methods available to reconstruct the fracture process, and thus emerges the requirement of some supporting parallel methods, which could be implemented very independently to reconstruct the fracture process.

A novel supporting method using an IR traceable liquid component to detect the change in concentration of the component during a rupture process, which could be used to eventually trace back the process of rupture, was thought of. The method was devised to relate the change in concentration of the component as a function of tearing energy, the latter in turn related to the speed of deformation of the matrix of the ruptured material. This innovative idea is neither described theoretically nor experimentally. This is where a big scope of a scientific investigation opened up.

Based on such an enormous scope, the objective of the present article was to find a means to study fractography by reverse engineering a fracture process by relatively quantifying the extraction of a used grade of liquid matter (in this case a paraffin process oil) from the rubber matrix to the fractured surface. It was thought that by observing the change in concentration of the oil over a defined fractured area after the incidence of rupture under varied specific loading conditions, a way may be paved to reverse engineer the fractrographic process by reconstructing the relative speed with which a matrix might have deformed before the incidence of a fracture finally giving way to a very fast rupture.

## 2. Materials and Methods

NR of grade SVR CV-60, Vietnam was compounded with paraffin oil Tudalen 3912B, H&R Group, Hamburg, Germany, along with other essential compounding ingredients. The compounding was done in an internal mixer (Brabender Plastograph, Brabender GmbH & Co. KG, Duisburg, Germany) at a rotor speed of 65 rpm and the mixing temperature was maintained at around 65 °C. At a mixer void volume of 50 cm^3^, mixing was done at a fill factor of 0.85. After mixing, each batch was cured at 150 °C under a pressure of 200 kPa in a hydraulic press (LabEcon 300, Fontijne Presses, Delft, The Netherlands) into a sheet of size 125 × 125 × 2 mm^3^ at optimum cure time, *t_90_*. The optimum cure time for each sample, which was about 8 min at 150 °C was determined in a moving die rheometer (MonTech MDR 3000 Basic, GmbH, Buchen, Germany). The compositions in terms of compounding ingredients for the two prepared batches are shown in Table 1.

The sample designation for the various initial oil concentrations and deformation speeds are shown in Table 2.

SENT tensile samples with dimension 20 × 10 × 2 mm^3^ (length, *L_0_* × width, *Q* × thickness, *B*) were punched out from the cured sheets and notched by initial length a = 1 mm from the edge in the positive direction of x axis, the edge taken as *x* = 0. (Figure 2) The notching was realized with a metal blade set to notch a predetermined length using indigenous equipment. After notching, each sample was fixed in a clamping system by using cylindrical shoulders on both sides to avoid any slippage of sample from the clamps during analyses. A uniaxial tensile test for each clamped, prepared rubber sample at an ambient temperature of 23 °C was done at two different deformation speeds of 10 and 100 mm·min^−1^ in a universal tensile testing machine (Testometric, Rochdale, UK). Three samples from each of the two compounded and cured rubber compositions were analyzed per deformation speed. The tearing energy, *T* was calculated by using Equation (4) in the force, F, versus deformation, l plot.

(4) $$T\propto \sum_{i=0}^{n}(F_{i}\cdot \Delta l_{i})/A$$

where, *A* = (*Q* − *a*)*B* represents the area generated during the tearing process leading to rupture.

The values of *F* and *l* were obtained from zero deformation up to deformation at rupture observed in the direction of uniaxial tensile loading.

It is important to mention here that when compared to Equation (3), which was used by earlier authors to calculate the tearing energy using J integral, the right hand side of Equation (4) produced proportionality with tearing energy and was not the absolute value. However, in the present work only a comparison of tearing energies between the different samples was sufficient, and so Equation (4) was adopted to calculate the tearing energy.

**Figure 3:**
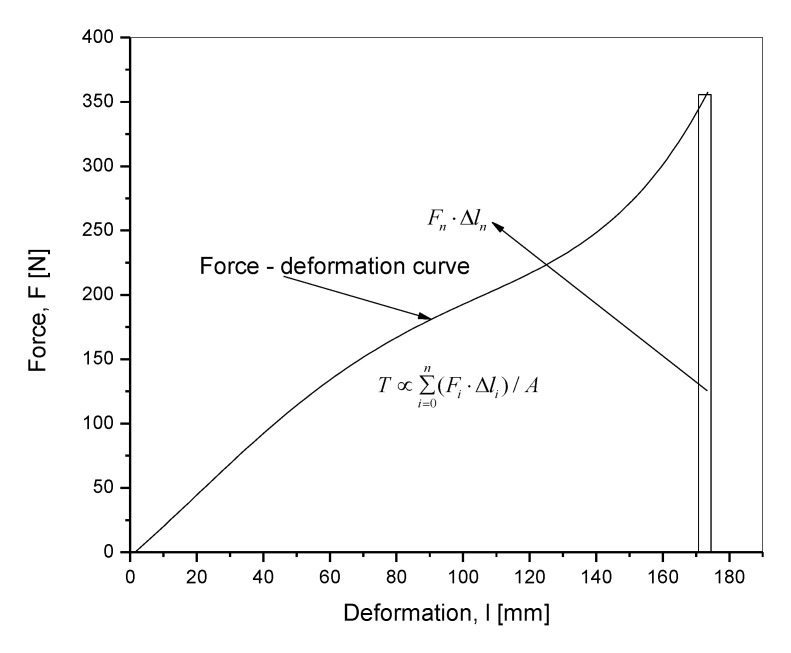
A typical force, *F*—deformation, *l* diagram for the calculation of the tearing energy.

Uniaxial deformation of the sample in the direction of clamp separation was considered as the deformation, and in the plot was taken as the independent parameter shown on the x axis. A representative force, *F*—deformation, *l* diagram to show the calculation of tearing energy is shown in Figure 3.

**Figure 4:**
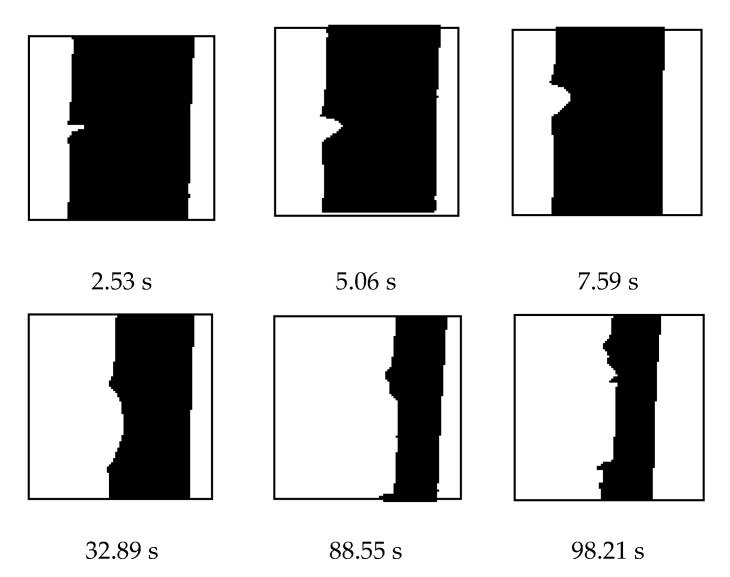
Photos taken with time designated as second, s for a sample to show that the sample suddenly ruptured after withstanding a long deformation.

The progress of the tensile testing from zero elongation to final rupture is arrested in Figure 4, It shows photographs captured by an optical camera, (DMK, 42BUC03 monochrome industrial camera, The Imaging Source Europe GmbH, Bremen, Germany), from some intermediate stage up to the time just prior to rupture with the corresponding time from the beginning of the experiment shown with each photo. It signifies that the sample kept on deforming in uniaxial elongation under a constant deformation speed with only crack blunting and no crack propagation; nearing the end of the process, the crack suddenly propagated very fast to cause rupture.

ATR FT-IR studies of the newly created fracture surfaces after rupture of tensile specimens at ambient temperature of 25 °C were done in the wavenumber range of 4000 to 650 cm^−1^ using an infrared spectrometer (––Nicolet iS5, Thermo Fisher Scientific, Madison, WI, USA). The spectra were obtained at a resolution of 2 cm^−1^ using a germanium crystal. Characteristic absorbance peak height of the oil at (723 cm^−1^) [22] was determined at a distance, as = 3 mm from the edge of the sample (the edge taken as 0 from the positive direction of x axis as is shown in Figure 2), on the two newly generated surfaces. For quantifying the peak heights, the spectrum for each sample was modified with an algorithm of baseline subtraction [22,23,24,25].

For each batch, random samples of three were taken and the median value of the three readings varying within only a negligible limit was reported as the result.

Finally, a percent change in tearing energy, T and also percent change in IR absorbance peak height, *A* at any fixed initial oil concentration at a time were calculated according to Equations (5) and (6).

*% change in T = [(T at 10 mm·min^−1^ deformation speed − T at 100 mm·min^−1^ strain)]/T at 100 mm·min^−1^ deformation speed]·100* (5)

*% change in A = [(A at 10 mm·min^−1^ deformation speed − A at 100 mm·min^−1^ strain)]/A at 100 mm·min^−1^ deformation speed]·100* (6)

## 3. Results

**Figure 5:**
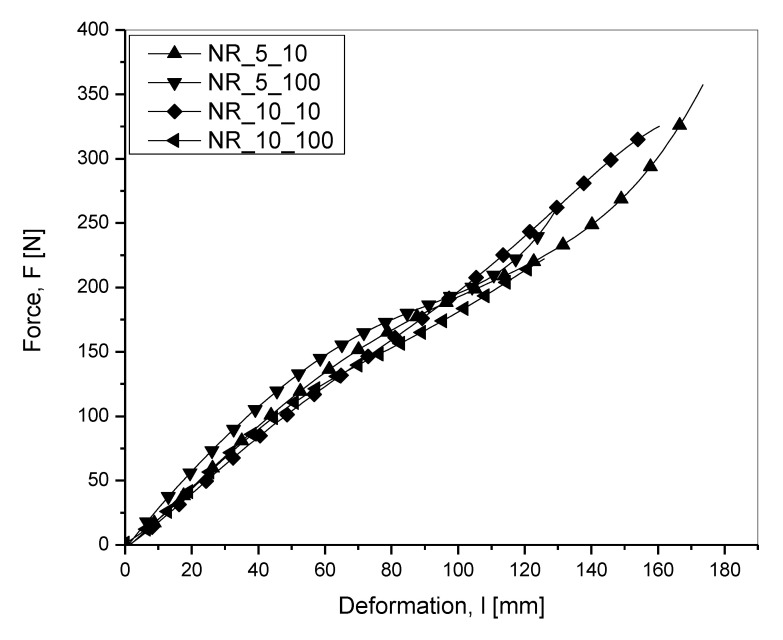
Superimposed force versus deformation curve for the two batches at two deformation speeds.

Shown in Figure 5 is the force versus deformation curves, one at random from three repetitions, and for each of the two batches, tested at two different deformation speeds from which tearing energy was calculated.

**Figure 6:**
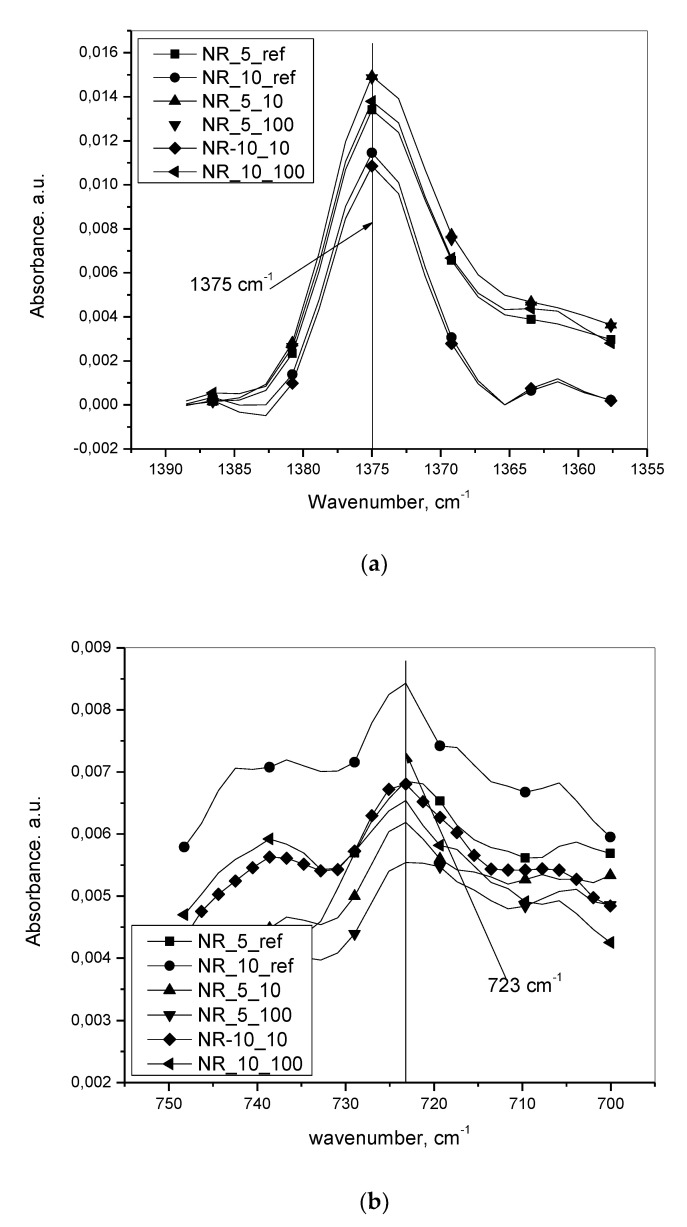
(**a**) the superimposed IR spectra after baseline fitting and subsequent subtraction for NR at 1375 cm^−1^ for the various initial oil incorporated batches at various deformation speeds. (**b**) Superimposed IR spectra after baseline fitting and subsequent subtraction for redistributed oil after rupture at 723 cm^−1^ for the various initial oil incorporated batches at various deformation speeds.

Figure 6a,b show the superimposed IR spectra after baseline fitting and subsequent subtraction for NR at 1375 cm^−1^ and for oil at 723 cm^−1^, one from three repetitions for each of the two compositions without deformation, and also for the two compositions at two different deformation speeds. For each of the batches, the spectrum was chosen that yielded after final calculation, the median value of the redistributed concentration of oil after rupture.

**Figure 7:**
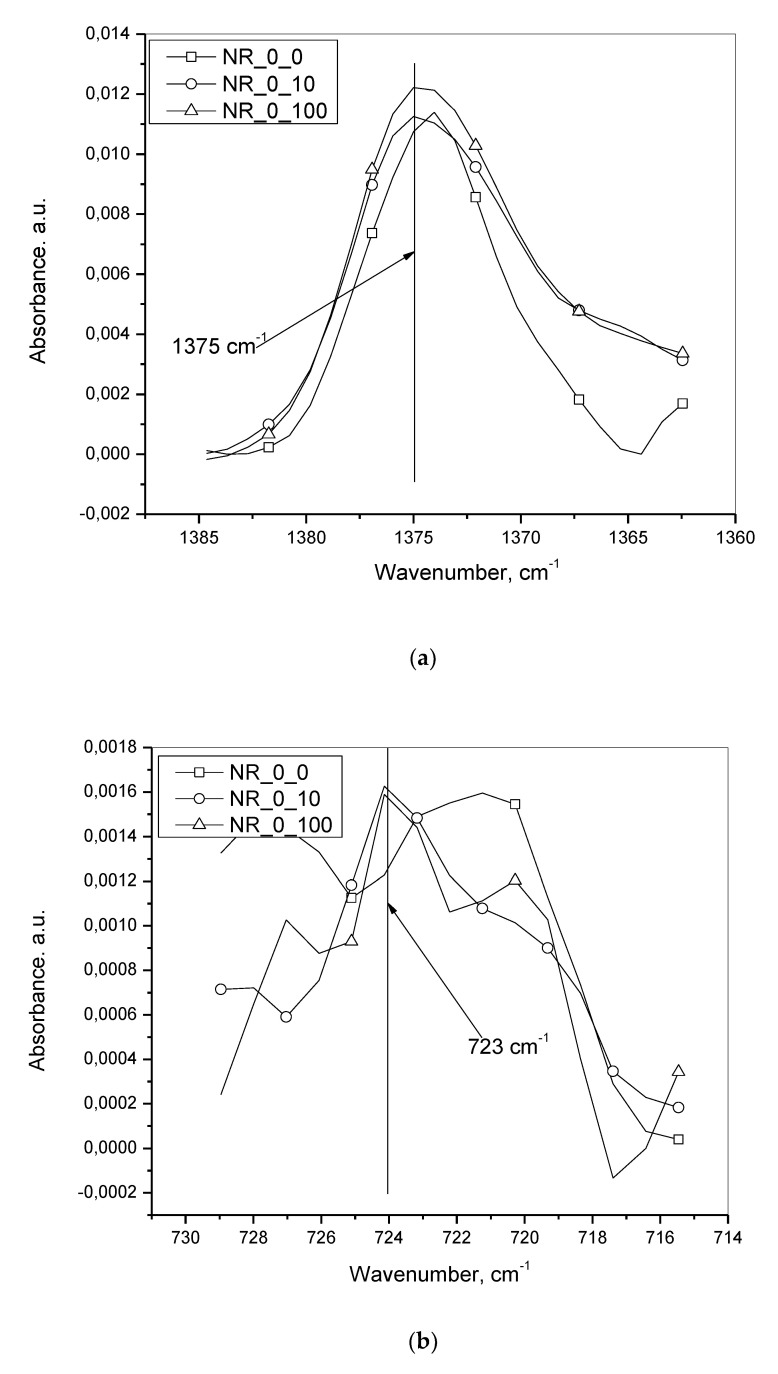
(**a**) the superimposed IR spectra after baseline fitting and subsequent subtraction for NR at 1375 cm^−1^ for zero oil incorporated batches at various deformation speeds. (**b**) Superimposed IR spectra after baseline fitting and subsequent subtraction for a component other than oil at 723 cm^−1^ for zero oil incorporated batches at various deformation speeds.

Figure 7a,b show the superimposed IR spectra after baseline fitting and subsequent subtraction for NR at 1375 cm^−1^ and for a component other than oil at 723 cm^−1^, designated as NR_0_0, NR_0_10 and NR_0_100 (Table 2)_one from three repetitions for a composition without oil at zero, 10 and 100 mm·min^−1^ deformation speeds. For each of the speeds, that spectrum was chosen that yielded after final calculation, the median value of the concentration of a component which had an IR absorption peak at 723 cm^−1^.

The calculated values for the tearing energies in oil incorporated samples and corresponding IR absorbance peak heights at 723 cm^−1^ are shown in Table 3.

The calculated values of IR absorbance peak heights at 723 cm^−1^ without oil at zero, 10 and o100 mm·min^−1^ are shown in Table 4.

The percent change in tearing energy, as well as the percent change in IR absorbance peak height between 10 and 100 mm·min^−1^ deformation speed at defined initial oil concentrations are presented in Table 5.

The values of tearing energy and IR absorbance peak heights for calculating the percent (%) changes were taken from Table 3 and the calculations were done according to Equations (5) and (6).

**Figure 8:**
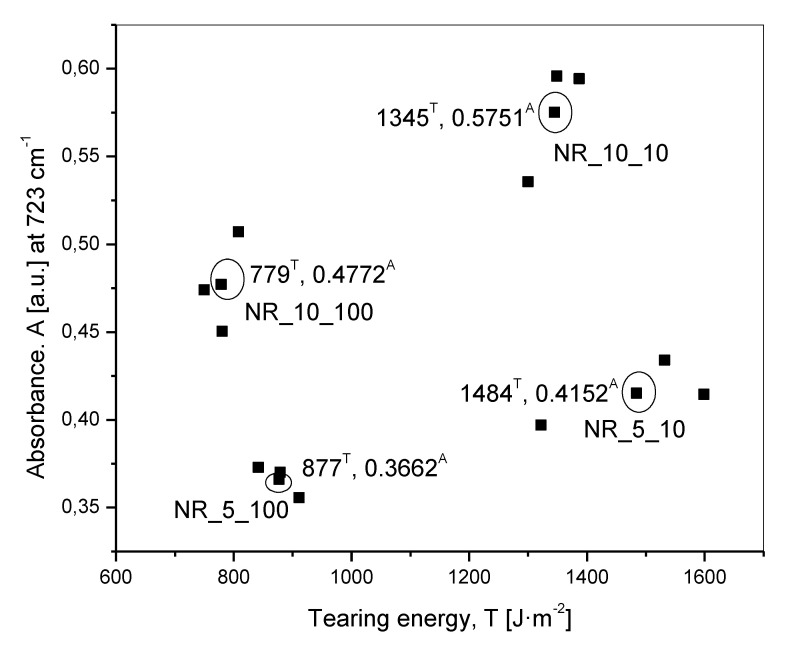
Scatter Plot of characteristic IR absorbance peak height for redistributed oil versus tearing energy.

To make the obtained results more comprehensible, data arrested in Table 3 are further illustrated in Figure 8, which shows the scatter plot of characteristic IR absorbance peak height versus tearing energy.

## 4. Discussion

The plot of IR absorbance peak height versus tearing energy—the tearing energy related to the deformation speed (Figure 8)—reflected some important trends, which may be summarized as follows:

(a) At a fixed initial oil concentration, the deformation speed determined the tearing energy. The tearing energy was found to be higher at a slower deformation speed of 10 mm·min^−1^ compared to that at a faster deformation speed of 100 mm·min^−1^.
(b) The IR absorbance peak height at 723 cm^−1^ at a given initial oil concentration was more at slower deformation speed. This was the most important observation pertaining to the title of the present work
(c) With an increase in initial oil concentration at any of the two defined deformation speeds, the absorbance peak height for oil at 723 cm^−1^ increased. However, as reflected in Table 3, the ratio of this peak height for 5 and 10 phr of oil did not show up as 5/10, simplified to 1/2 (0.5). This was probably due to the presence of a compound in the rubber compounding which had a peak at the same wavenumber of 723 cm^−1^. In effect, 1/2 (0.5) worked out to (1+p)/(2+p) = 0.7, which is greater than 0.5, where p was the additional peak height from the compound. To prove that another compound was adding to this absorbance peak height, a set of experiments with no oil at three defined deformation speeds of 0, 10 and 100 mm·min^−1^ were done, the result of which is shown in Table 4. It shows that this additional peak height was almost a constant, in effect proving that even in the presence of another compound having a peak at the same wavenumber as that of the oil, the scientific approach of the work was not affected, meaning that even without the subtraction of this peak height from the peak height of oil, the conclusions of the present work remained unaffected.
(d) At a given deformation speed, the tearing energy was higher for the lower initial concentration of the oil at 5 phr. This was attributed to the elongation at break, which was a little higher and at comparable elastic modulus (comparable slopes of all the force—displacement curves) an important parameter to determine the tearing energy.
(e) The percent changes in tearing energy and IR absorbance peak height between 10 and 100 mm·min^−1^ deformation speeds as calculated in Equations (5) and (6), respectively, at any defined initial oil concentration after the incidence of rupture revealed an interesting trend. Calculations show that for 5 and 10 phr of oil, the percent changes in tearing energy were 69.21 and 72.66 respectively and the percent changes in IR peak height were 13.37 and 20.54, respectively. The results revealed that the percent changes in all the cases were positive, proving that after redistribution, more of a redistributed concentration of oil was observed at higher tearing energy or at lower deformation speed.

Explanations for the trends will now be discussed in detail. It can be scientifically argued that at any of the two defined initial oil concentrations, wherever the tearing energy was more, the relative redistributed concentration of the oil after fracture on the defined fractured surface itself was always more. The higher redistributed concentration of oil was reflected as higher IR absorbance peak height on the predefined fractured surfaces after rupture.

The energy first went into elongating the test specimen in the direction of induced uniaxial deformation without increasing the length of the notch, as was very nicely captured by a high-speed camera. Finally, just before elongation at break, the length of the notch propagated very quickly, and the sample ruptured. Most of the energy absorbed during the process of elongation was the restoring potential energy, which allowed the rubbery material to snap back to almost its original dimensions after rupture. A part of the total energy absorbed during the elongation and the rupture process was lost as permanent deformation in the elongated species. Finally, a part also went into propagating the notch through successive fractures summing up to the final rupture.

The change in concentration of oil on the fractured surface to a higher value at higher tearing energy in the case of lower deformation speed was the manifestation of slower elongation, producing more viscous deformation of the rubbery matrix.

The observed facts need to be rigorously explained through the physics underlying polymer viscoelasticity, and may be a future scope of work

It may perhaps be said that at a slower deformation speed, the deformed rubber after rupture could not fully regain the mass, which was present earlier over the observed cross-sectional area, perpendicular to the axial deforming direction. However, the oil, which was also drawn away to some extent, regained more mass over rubber after rupture. This was reflected as higher redistributed oil concentration on the fractured surface compared to the test at higher deformation speed.

At higher deformation speed, the deformation in the rubbery phase was more elastic with less permanent deformation reflected as more regained mass of the rubber equivalent to less redistributed oil concentration on the fractured surface.

In the present work, since oil was also drawn away along with the rubber matrix during the elongation process, probably at a faster rate, so the redistributed local concentration of the oil after rupture, even after some regained mass was found to be less than that of the un-fractured samples. However, this may not be a universal rule because it depends upon the relative regaining of the masses between the oil and the rubber matrix over the observed cross-sectional area of fracture.

## 5. Conclusions

Single edge notched tensile samples (SENT) from cured natural rubber (NR) at two different processing paraffin oil initial concentrations of 5 and 10 phr, were subjected to uniaxial tensile loading at two different deformation speeds of 10 and 100 mm·min^−1^ for each initial oil concentration. The total energy to elongate the sample in uniaxial direction and to crack propagate a notch on the sample to rupture, per generated area during rupture (in the present context referred to as tearing energy), as a function of deformation speed at defined oil initial concentrations were determined. Further, predefined areas on the generated fractured surfaces after the incidence of rupture were subjected to spectroscopic investigation using attenuated total reflection (ATR) Fourier transform infrared (FT-IR) spectroscopy to quantify the redistribution of oil on the defined fractured surfaces.

Higher deformation speed decreased both, the tearing energy and the IR absorbance peak height response for the oil on the fractured surface after rupture.

The observed trends were explained in the results and discussion section of this article in terms of drawing away of oil and simultaneous viscous and elastic deformation of the rubber matrix during the process of uniaxial elongation and their relative recovery after rupture.

In the process of experimental observation was developed a novel approach verified through digital image correlation (DIC), validating the numerical calculation published by Kaliske et al. The expected highest deformation was just behind the notch tip, which was due to the vertical deformation, distributed above and below the tip, and with distance the deformation gradually decreased. Obviously, the concentrated forces around the crack tip and their orientation caused the crack opening, resulting in fracture. These forces were assumed to act on the solid, as well as on liquid matter in the matrix in a similar manner and in the same direction, the gradients oriented in the direction to the crack tip.

The present work based on experimental results produced different redistributed concentration of oil on ruptured surfaces of defined rubber specimens as function of speed of deformation, starting from the same initial oil concentration. From this concept the reverse methodology can be applied, which is the most important outcome of the present work by comparing the relative final concentration of oil at ruptured surfaces, between two or more cured rubber specimens all having the same initial oil concentration, the comparison done by using a simple but novel IR technique, the relative speed of deformation which brought about such a rupture can be assessed, thus paving a novel way to reverse engineer a fractographic process.

Future work aims at introducing an additional deformation speed over the two existing ones, targeted to find a mathematical relation between the redistributed oil concentration studied through IR spectroscopy and the tearing energy studied by using tensile testing.

## Figures and Tables

**Table 1:** Composition of the prepared batches.

Compounding Ingredients	phr *
NR (SVR CV-60)	100.00
Zinc oxide (ZnO)	3.00
Stearic acid	1.00
Carbon black, N-330	50.00
Paraffin Oil, Tudalen 3912B	5.00 (Batch-1) 10.00 (Batch-2). 0.00 (Batch-3)
Rubber Accelerator, CBS **	2.50
Sulphur	1.70

* parts per hundred rubber by weight; ** N-Cyclohexyl-2-benzothiazole sulphenamide.

**Table 2:** Sample designation of the various batches at various initial oil concentrations and two different deformation speeds.

Sample Designation	Initial Oil Concentration, phr	Deformation Speed, mm·min^−1^
NR_5_ref *	5.00	0
NR_10_ref	10.00	0
NR_5_10	5.00	10
NR_5_100	5.00	100
NR_10_10	10.00	10
NR_10_100	10.00	100
NR_0_0	0.00	0
NR_0_10	0.00	10
NR_0_100	0.00	100

* reference sample not subjected to any deforming strain.

**Table 3:** Tearing energy and corresponding IR absorbance peak height for redistributed oil.

Sample Designation	Tearing Energy. J·m^−2^	IR Absorbance at 1375 cm^−1^(NR). as Obtained after Baseline Subtraction	IR Absorbance at 723 cm^−1^ (oil). as Obtained after Baseline Subtraction	IR Absorbance at 723 cm^−1^ (oil) Calculated against Normalized Peak at 1375 cm^−1^ **
NR_5_0	not applicable	0.0136	0.0074	0.5456
		0.0132	0.0068	0.5168
		0.0134	0.0068	0.5109
		0.0134 (±0.0001) *	0.0070 (±0.0003)	0.5244 (±0.0151)
NR_10_0	not applicable	0.0115	0.0084	0.7363
		0.0109	0.0086	0.7967
		0.0115	0.0079	0.6902
		0.0113 (±0.0003)	0.0083 (±0.0003)	0.7411 (±0.0436)
NR_5_10	1322	0.0146	0.0058	0.3970
	1599	0.0149	0.0062	0.4145
	1532	0.0150	0.0065	0.4340
	1484 (±118)	0.0148 (±0.0001)	0.0062 (±0.0003)	0.4152 (±0.0151)
NR_5_100	842	0.0149	0.0055	0.3729
	911	0.0151	0.0054	0.3556
	879	0.0150	0.0056	0.3701
	877 (±28)	0.0150 (±0.0001)	0.0055 (±0.0001)	0.3662 (±0.0076)
NR_10_10	1387	0.0115	0.0068	0.5943
	1349	0.0109	0.0065	0.5958
	1300	0.0115	0.0061	0.5356
	1345 (±35)	0.0113 (±0.0003)	0.0065 (±0.0003)	0.5751 (±0.0093)
NR_10_100	780	0.0135	0.0061	0.4504
	808	0.0134	0.0068	0.5072
	750	0.0138	0.0065	0.4740
	779 (±23)	0.0136 (±0.0002)	0.0065 (±0.0003)	0.4772 (±0.0233)

* The values within parenthesis are the standard deviations calculated over a population of three samples with the arithmetic mean shown outside the parenthesis; ** The IR absorbance values for redistributed oil in this column are taken for all subsequent discussion.

**Table 4:** Tearing energy and corresponding IR absorbance peak height at 723 cm^−1^ for a component other than oil.

Sample Designation	IR Absorbance at 1375cm^−1^ (NR). as Obtained after Baseline Subtraction	IR Absorbance at 723 cm^−1^ (without oil). as Obtained after Baseline Subtraction	IR Absorbance at 723 cm^−1^ (without oil) Calculated against Normalized Peak at 1375 cm^−1^ **
NR_0_0	0.0101	0.0020	0.1972
	0.0114	0.0016	0.1400
	0.0128	0.0016	0.1211
	0.0115 (±0.0011) *	0.0017 (±0.0002)	0.1528 (±0.0323)
NR_0_10	0.0113	0.0016	0.1445
	0.0113	0.0021	0.1662
	0.0132	0.0017	0.1313
	0.0119 (±0.0009)	0.0018 (±0.0002)	0.1473 (±0.01439)
NR_0_100	0.0122	0.0016	0.1302
	0.0127	0.0014	0.1121
	0.0124	0.0019	0.1542
	0.0124 (±0.0002)	0.0016 (±0.0002)	0.1322 (±0.0172)

* The values within parenthesis are the standard deviations calculated over a population of three samples with the arithmetic mean shown outside the parenthesis; ** The IR absorbance values for redistributed oil in this column are taken for all subsequent discussion.

**Table 5:** Percent changes in tearing energy and IR absorbance peak height between 10 and 100 mm·min^−1^ deformation speed at defined initial oil concentration.

% Change in Tearing Energy		% Change in IR Absorbance Peak Height	
defined for 5 phr of oil	defined for 10 phr of oil	defined for 5 phr of oil	defined for 10 phr of oil
69.21	72.66	13.37	20.54
